# Phenformin Induces Cell Cycle Change, Apoptosis, and Mesenchymal-Epithelial Transition and Regulates the AMPK/mTOR/p70s6k and MAPK/ERK Pathways in Breast Cancer Cells

**DOI:** 10.1371/journal.pone.0131207

**Published:** 2015-06-26

**Authors:** Zhao Liu, Lidong Ren, Chenghao Liu, Tiansong Xia, Xiaoming Zha, Shui Wang

**Affiliations:** Department of Breast Surgery, the First Affiliated Hospital of Nanjing Medical University, Nanjing, China; Virginia Commonwealth University, UNITED STATES

## Abstract

Breast cancer remains a world-wide challenge, and additional anti-cancer therapies are still urgently needed. Emerging evidence has demonstrated the potent anti-tumor effect of biguanides, among which phenformin was reported to potentially be a more active anti-cancer agent than metformin. However, little attention has been given to the role of phenformin in breast cancer. In this study, we reveal the role of phenformin in cell death of the MCF7, ZR-75-1, MDA-MB-231 and SUM1315 breast cancer cell lines. The respective IC_50_ values of phenformin in MCF7, ZR-75-1, MDA-MB-231 and SUM1315 cells were 1.184±0.045 mM, 0.665±0.007 mM, 2.347±0.010 mM and 1.885±0.015 mM (mean± standard error). Phenformin induced cell cycle change and apoptosis in breast cancer cells via the AMPK/mTOR/p70s6k and MAPK/ERK pathways. Interestingly, phenformin induced MET (mesenchymal-epithelial transition) and decreased the migration rate in breast cancer cell lines. Furthermore, our results suggest that phenformin inhibits breast cancer cell metastasis after intracardiac injection into nude mice. Taken together, our study further confirms the potential benefit of phenformin in breast cancer treatment and provides novel mechanistic insight into its anti-cancer activity in breast cancer.

## Introduction

Breast cancer, the most frequently diagnosed carcinoma in females and the second leading cause of cancer death in women, is a heterogeneous disease with various pathological entities[1]. Despite the efficacy of many anti-cancer agents and the improved disease-free survival and overall survival of breast cancer patients, some patients still succumb to this disease[2]. Therefore, additional anti-cancer therapies are still needed.

Biguanides, such as metformin and phenformin, are commonly used as therapeutics for type 2 diabetes[3]. Patients with diabetes who were treated with metformin experienced a 31% reduction in the overall relative risk of cancer incidence and cancer-related mortality compared with those treated with other therapeutics[4]. Moreover, retrospective studies have reported an association between metformin use and improved cancer-related mortality[5]. These anti-tumor effects were first described by Lugaro and Giannattasio in 1968[6]. Since then, the anti-tumor activity of biguanides in animal models and cell lines has been reported by many other authors. However, studies on cancer prevention and treatment with biguanides have mainly focused on metformin [7].

As a therapeutic for diabetes, phenformin use has been limited to relatively few countries because of an increased incidence of phenformin-associated lactic acidosis in elderly patients with renal failure compared with metformin treatment [8]. Nevertheless, phenformin was more active against tumor cells than metformin [9]. Phenformin was reported to be much more potent than metformin as an anti-tumor agent, apparently because metformin requires an organic cation transporter (OCT) to enter tumor cells [10]. Moreover, it was recently reported that supplementation of 2-deoxyglucose with phenformin may avoid the risk of lactic acidosis. Therefore, phenformin should be re-examined as a potential agent for cancer prevention and treatment [11].

The activation of AMPK(AMP-activated protein kinase) signaling and the attenuation of ERK (extracellular signal-regulated kinase) signaling are known to contribute to the anti-tumor effects of metformin [12]. Furthermore, metformin reversed epithelial-mesenchymal transition (EMT) in human breast cancer cells [13]. Phenformin inhibited the growth of breast cancer cells by activating AMPK [14]. However, the other effects of phenformin and its mechanism of action in breast cancer are currently unknown. In this study, we utilized the MCF7, ZR-75-1, MDA-MB-231 and SUM1315 cell lines to ascertain the anti-tumor effects of phenformin in breast cancer cell lines of different genetic backgrounds and to further explore the underlying molecular mechanism of the action of this drug. Migration assays and an intracardiac injection mouse model (BALB/c nude mice) were used to elucidate the role of phenformin in breast cancer metastasis.

## Materials and Methods

### Ethics statement

All the animal protocols were approved by the Institutional Animal Care and Use Committee of Nanjing Medical University. All the animal experiments were monitored by the Department of Laboratory Animal Resources of Nanjing Medical University.

### Cell culture

The human breast cancer cell lines MCF7, ZR-75-1, and MDA-MB-231 were obtained from American Tissue Culture Collection (ATCC). The human breast cancer cell line SUM1315 was kindly provided by Dr. Stephen Ethier University of Michigan (http://www.cancer.med.umich.edu/breast_cell/Production/index.html). All the cell lines were cultured in DMEM (Wisent, Nanjing, China) supplemented with 10% fetal bovine serum (FBS; Wisent, Nanjing, China) and maintained in a humidified incubator at 37°C with CO_2_. Cells were split upon reaching 85% confluence.

### Colorimetric CCK-8 assay

Cells (5,000) were plated in wells of a 96-well plate containing different concentrations of phenformin (0mM, 0.5 mM, 1 mM, 2 mM or 4 mM). The cells were incubated in a humidified incubator at 37°C with CO_2_ for 24 hours. Two hours before the end point, 10 μl of CCK-8 solution was added to each well, and the cells were incubated at 37°C for 2 more hours. The absorbance was then measured at 450 nm using an automated microplate reader (Tecan, 5082 Grodig, Austria); each experiment was repeated three times. The percent growth inhibition was calculated using the following formula: (OD of the control- OD of the experimental sample)/OD of the control×100%. The half-maximum growth inhibitory concentration (IC_50_) was taken as the concentration that elicited 50% inhibition of cell viability. Linear regression was performed after log transformation of the X axes using GraphPad Prism 5 to calculate the IC50 values.

### Cell cycle analysis

MCF7, ZR-75-1, MDA-MB-231 and SUM1315 cells were exposed to different concentrations of phenformin (the IC_50_ of each cell line: 1.184 mM, 0.665 mM, 2.347 mM, and 1.885 mM, respectively) for 24 hours; then, the cells were collected and fixed with 70% ethanol overnight at 4°C. The fixed cells were washed with PBS, stained with 10 μg/ml propidium iodide (PI); (Carlsbad, CA, USA) in the presence of 5 μg/ml RNase A and analyzed for fluorescence using a FACSAria flow cytometer (Becton Dickinson, San Jose, CA, USA).

### Apoptosis analysis

MCF7, ZR-75-1, MDA-MB-231 and SUM1315 cells were exposed to different concentrations of phenformin (the IC_50_ of each cell line: 1.184 mM, 0.665 mM, 2.347 mM, and 1.885 mM respectively) for 24 hours. The cells were then washed twice with PBS at 4°C, resuspended in staining solution containing propidium iodide (PI) and Annexin V-FITC (Carlsbad, USA) for 15 minutes at 4°C and analyzed with a flow cytometer (Becton Dickinson, USA).

### Western blotting analysis

MCF7, ZR-75-1, MDA-MB-231 and SUM1315 cells were exposed to different concentrations of phenformin (the IC_50_ of each cell line: 1.184 mM, 0.665 mM, 2.347 mM, and 1.885 mM, respectively) for 24 hours. After treatment, the cells were scraped from the plates, rinsed with PBS and lysed in cell lysis buffer containing protease inhibitor and phosphatase inhibitors to obtain total protein extracts. The protein concentrations were determined using a BCA protein assay (ThermoScientific, Tewksbury, USA), and equal amounts of protein (60 μg) were separated by SDS-PAGE and transferred to PVDF membranes. The membranes were incubated with the following primary antibodies: anti-p-AMPK, anti-AMPK, anti-p-mTOR, anti-mTOR, anti-p-p70s6k, anti-p70s6k, anti-p-ERK, anti-ERK, anti-cyclin D1, anti-cleaved caspase 3 and anti-E-cadherin (Cell Signaling Technology; Danvers, MA, USA); anti-vimentin (Abcam; Cambridge, MA, USA.); and anti-GAPDH (Bioworld; Nanjing, China). Anti-rabbit and anti-mouse secondary antibodies were obtained from Bioworld (Nanjing, China). The antibodies were diluted according to the manufacturer’s instructions. The secondary antibodies used for detection were conjugated with horseradish peroxidase (HRP). Protein were visualized with the SuperSignal West Femto Maximum sensitivity substrate kit (Thermo Scientific, Logan, USA). The signals of protein were detected by FluorChem E System (Proteinsimple, San Jose, USA). The density of each band was quantified using Photoshop and plotted using GraphPad Prism 5. The levels of the phosphorylated protein expressions were normalized to those of the corresponding total protein; the expression levels of all the other targeted proteins were normalized to GAPDH expression.

### Cell migration assay

Cell migration assays were performed in 24-well Boyden Chambers with 8-μm-pore polycarbonate membranes (BD Bioscience, Franklin Lakes, NJ, USA). After incubation with phenformin (2.347 mM) for 24 hours, MDA-MB-231 cells were seeded at 25,000 cells per chamber in the upper chamber, which contained serum-free medium. The lower chamber contained medium with 10% FBS. After incubation for 16 hours, the cells on the upper surface of the membrane were removed, and those on the lower surface of the chamber were stained with crystal violet and counted using a microscope.

### Animal experiments

Four-to five-week-old female BALB/c nude mice were purchased from the Model Animal Research Center of Nanjing University. An intracardiac injection model was used to evaluate the role of phenformin in the regulation of breast cancer metastasis. MDA-MB-231 cells were tagged with luciferase. Briefly, the luciferase-tagged MDA-MB-231 cells (1x10^5^ cells/mouse) were injected into the left cardiac ventricle of anesthetized mice. Mice were anesthetized with 2% isoflurane using an inhalational anesthesia system. After the injections, a total of twenty mice were separated into the control and phenformin groups (10 mice per group). The control group’s drinking water was replaced with 5% sucrose, and the phenformin group’s drinking water was replaced with 5% sucrose containing phenformin (300 mgkg^-1^). Four weeks after the intracardiac injection, mice were injected intraperitoneally with 150mgkg^-1^ D-luciferin. The development of metastasis was monitored using a Xenogen in vivo imaging system (IVIS) with a cooled CCD camera. (Perkin-Elmer, Waltham, MA, USA). The utilized drug concentration was chosen based on data from the literature [7]. Animals were sacrificed using pure carbon dioxide.

### Statistical analysis

All the data for the different experimental groups are expressed as the mean±SEM, and the data were obtained from at least three independent experiments. The significant differences between the control and experimental groups were determined using Student’s t test. The Wilcoxon rank sum test was used to determine the significant differences in total flux between the control and phenformin treated animals. All the statistical analyses were performed using STATA 11 (StataCorp LP, College Station, TX, USA.). Differences with p<0.05 were considered statistically significant.

## Results

### Phenformin induces cell death of breast cancer cells

**Fig 1:**
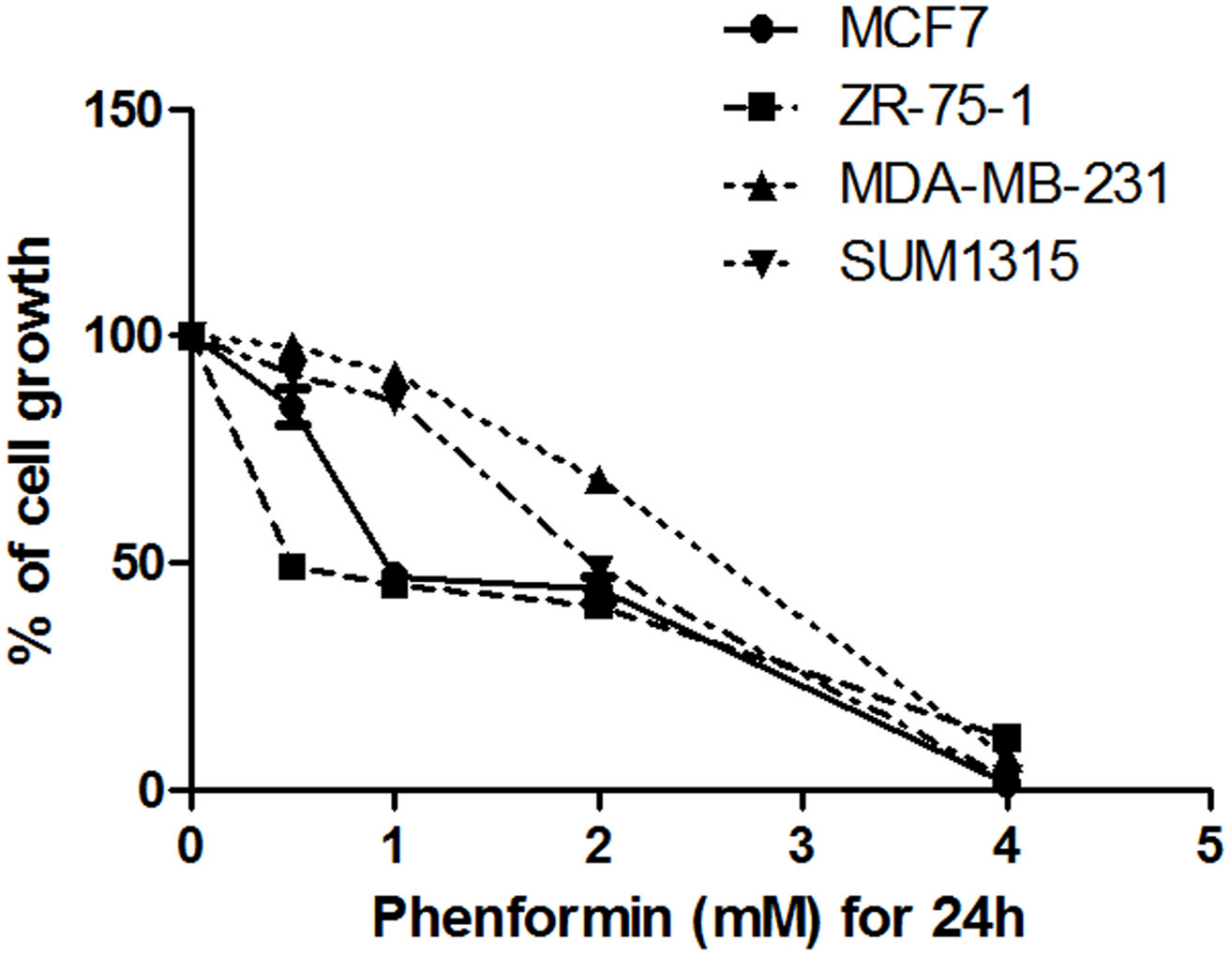
Phenformin induces cell death of breast cancer cell. Cells were grown for 24 hours in the presence of the indicated concentration of phenformin. CCK-8 assays were performed to evaluate cell viability. The absorbance was measured at 450 nm using an automated microplate reader. The percent growth inhibition was calculated using the following formula: (OD of the control-OD of the experimental sample)/OD of the control×100%.

To evaluate the activity of phenformin in breast cancer cell lines of different genetic backgrounds, two ERα-positive breast cancer cell lines (MCF7, ZR-75-1) and two ERα-negative breast cancer cell lines (MDA-MB-231, SUM1315) were used. The CCK-8 assay was used to detect the inhibitory effect of phenformin on the different breast cancer cell lines. Increasing doses of phenformin resulted in a dose-dependent cell death of all 4 breast cancer cell lines. MCF7 and ZR-75-1 cells were more sensitive to phenformin, whereas MDA-MB-231 and SUM1315 cells were less sensitive to phenformin (Fig 1). The respective IC_50_ values of phenformin in the MCF7, ZR-75-1, MDA-MB-231 and SUM1315 cells were 1.184±0.045 mM, 0.665±0.007 mM, 2.347±0.010 mM and 1.885±0.015 mM (mean± standard error).

### Phenformin induces an increase in the percentage of cells in G1 and inhibits ERK signaling in breast cancer cells

**Fig 2:**
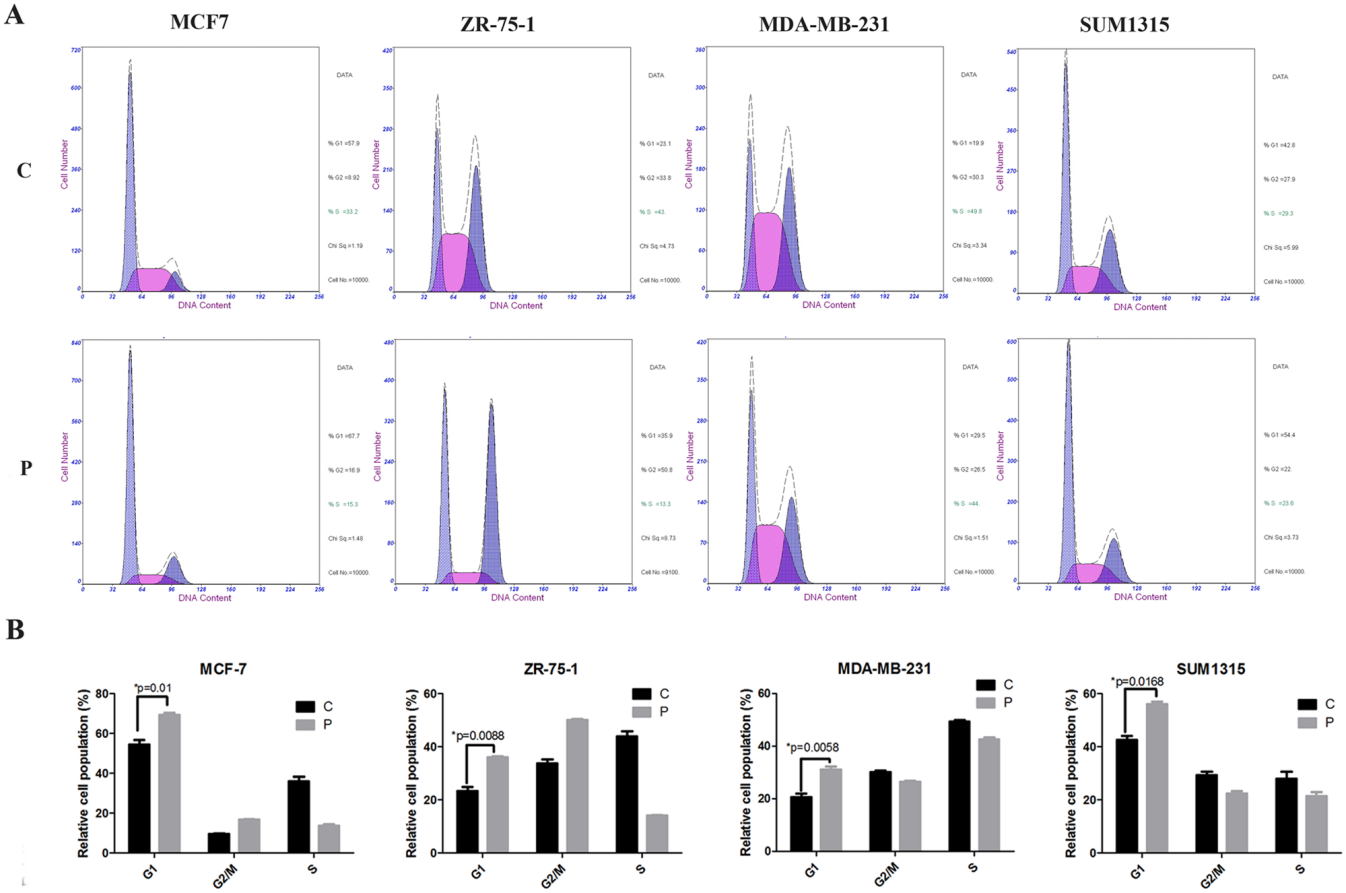
Phenformin induces an increase in the percentage of cells in G1 in breast cancer cells. (A) MCF7, ZR-75-1, MDA-MB-231 and SUM1315 cells were treated with 1.184 mM, 0.665 mM, 2.347 mM, and 1.885 mM phenformin (the IC_50_ of each cell lines) respectively for 24 hours. Control cells were treated with solvent (42.9% DMSO in DMEM). The cells were fixed with ethanol, stained with PI and analyzed by flow cytometry. (B) The percentages of cells with a DNA content consistent with each phase of the cell cycle were plotted. The data are presented as the mean±SEM of three replicates per group. Asterisks indicate significant differences at p<0.05 by Student’s t test. Phenformin treated cells were labeled as P and control cells were labeled as C.

**Fig 3:**
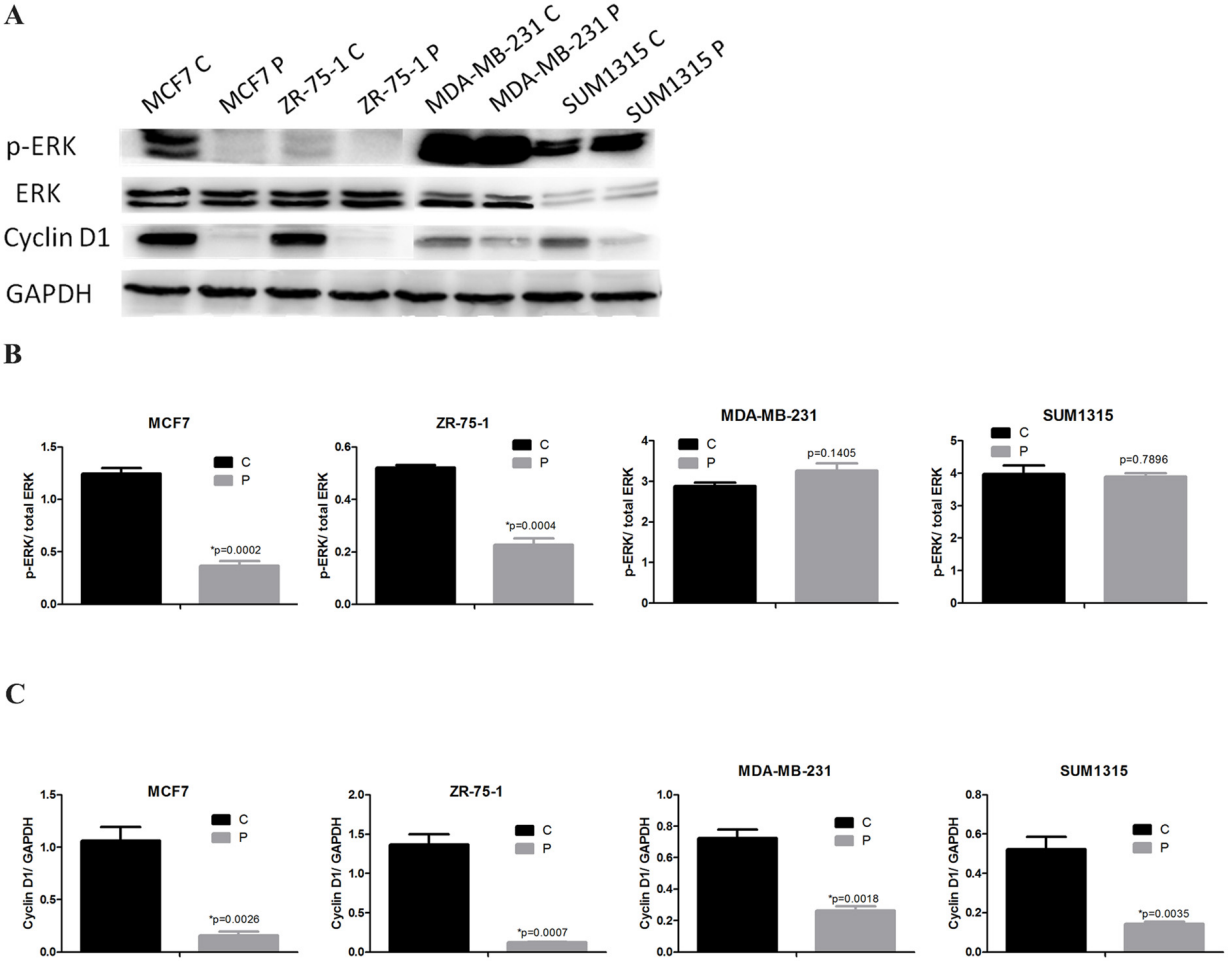
Phenformin downregulates cyclin D1 expression and inactivates the MAPK/ERK pathway. (A) MCF7, ZR-75-1, MDA-MB-231 and SUM1315 cells were treated with or without phenformin for 24 hours. Cell extracts were analyzed by western blotting to detect the expression of cyclin D1, p-ERK, ERK and GAPDH. (B) Western blotting ratio analysis of p-ERK to ERK, (C) Cyclin D1 to GAPDH. The data are presented as the mean±SEM of three replicates per group. Asterisks indicate significant differences at p<0.05 by Student’s t test. Phenformin-treated cells were labeled as P and control cells were labeled as C.

To examine whether the growth inhibitory effect of phenformin on breast cancer cells was partly due to cell cycle change, we performed cell cycle analysis using PI staining. MCF7, ZR-75-1, MDA-MB-231 and SUM1315 cells were exposed to 1.184 mM, 0.665 mM, 2.347 mM, and 1.885 mM phenformin, (the IC_50_ of each cell line) respectively for 24 hours. The percentage of phenformin-treated MCF7, ZR-75-1, MDA-MB-231 and SUM1315 cells in G1 significantly increased to 67.7% (p = 0.01), 35.9% (p = 0.0088), 29.5% (p = 0.0058) and 54.4% (p = 0.0168), respectively (Fig 2). Consistent with the CCK8 assay results, the lowest percentage of cells in G1 was observed in MDA-MB-231 cells. To further confirm the increased cells in G1 induced by phenformin, we performed western blotting assays to detect cyclin D1 expression. Notably, significantly lower levels of cyclin D1 protein were observed in all four breast cancer cell lines treated with phenformin (Fig 3), which suggested that phenformin might regulate the cell cycle of breast cancer cells by modulating cyclin D1. Interestingly, the regulation of ERK phosphorylation by phenformin diverged between the ERα-positive and ERα-negative breast cancer cell lines. The p-ERK levels in MCF7 and ZR-75-1 cells decreased significantly upon phenformin treatment. In contrast, p-ERK levels did not change in MDA-MB-231 or SUM1315 cells after treatment with phenformin (Fig 3).

### Phenformin promotes breast cancer cell apoptosis

**Fig 4:**
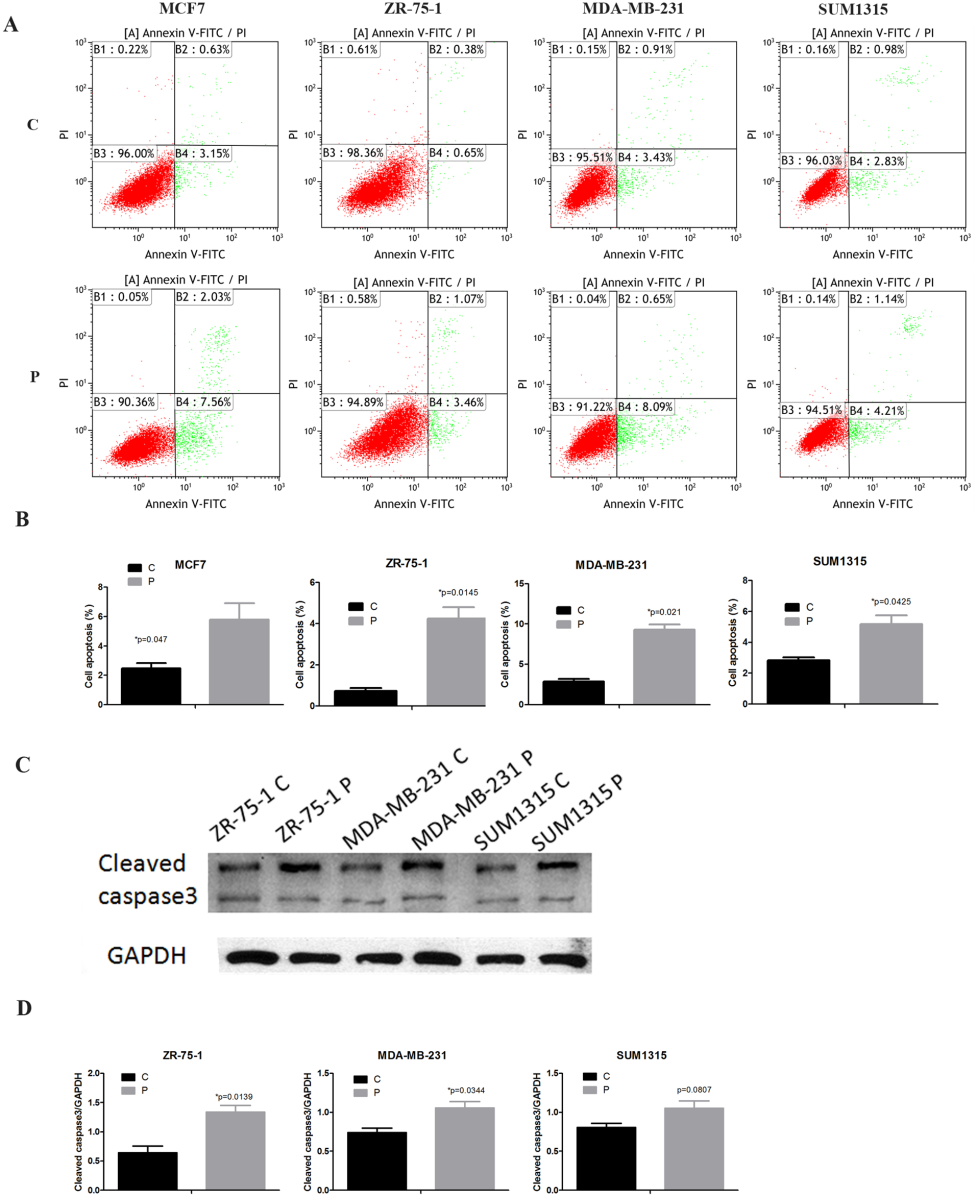
Phenformin promotes breast cancer apoptosis. (A) MCF7, ZR-75-1, MDA-MB-231 and SUM1315 cells treated with or without phenformin were double-stained with Annexin V and PI, and then analyzed by flow cytometry. (B) The percentages of Annexin V^+^/PI^-^ cells(early apoptosis) were plotted. (C) Cell extracts were analyzed by western blotting to detect the expression of cleaved caspase 3 and GAPDH. (D) Expression ratio of cleaved caspase 3 to GAPDH. The data are presented as the mean±SEM of three replicates per group. Asterisks indicate significant differences at p<0.05 by Student’s t test. Phenformin-treated cells were labeled as P and control cells were labeled as C.

Breast cancer cells treated with phenformin were double-stained with Annexin V and PI, and the cellular apoptosis rates were determined by flow cytometry. In cells treated with phenformin, Annexin V^+^/PI^-^ (early apoptosis) subpopulation significantly increased. Compared with control cells, the early apoptosis rates of phenformin-treated MCF7, ZR-75-1, MDA-MB-231 and SUM1315 cells increased to 7.56% (p = 0.043), 3.46% (p = 0.0145), 8.09% (p = 0.021) and 4.21% (p = 0.0425), respectively (Fig 4A and 4B). To further confirm the induction of apoptosis by phenformin, we detected the expression of cleaved caspase 3 by western blotting. Interestingly, cleaved caspase 3 levels significantly increased in ZR-75-1 and MDA-MB-231 breast cancer cells treated with phenformin, suggesting that the caspase 3 pathway may be involved in phenformin induced apoptosis (Fig 4C and 4D).

### Activation of AMPK and inhibition of mTOR/p70s6k signaling by phenformin

**Fig 5:**
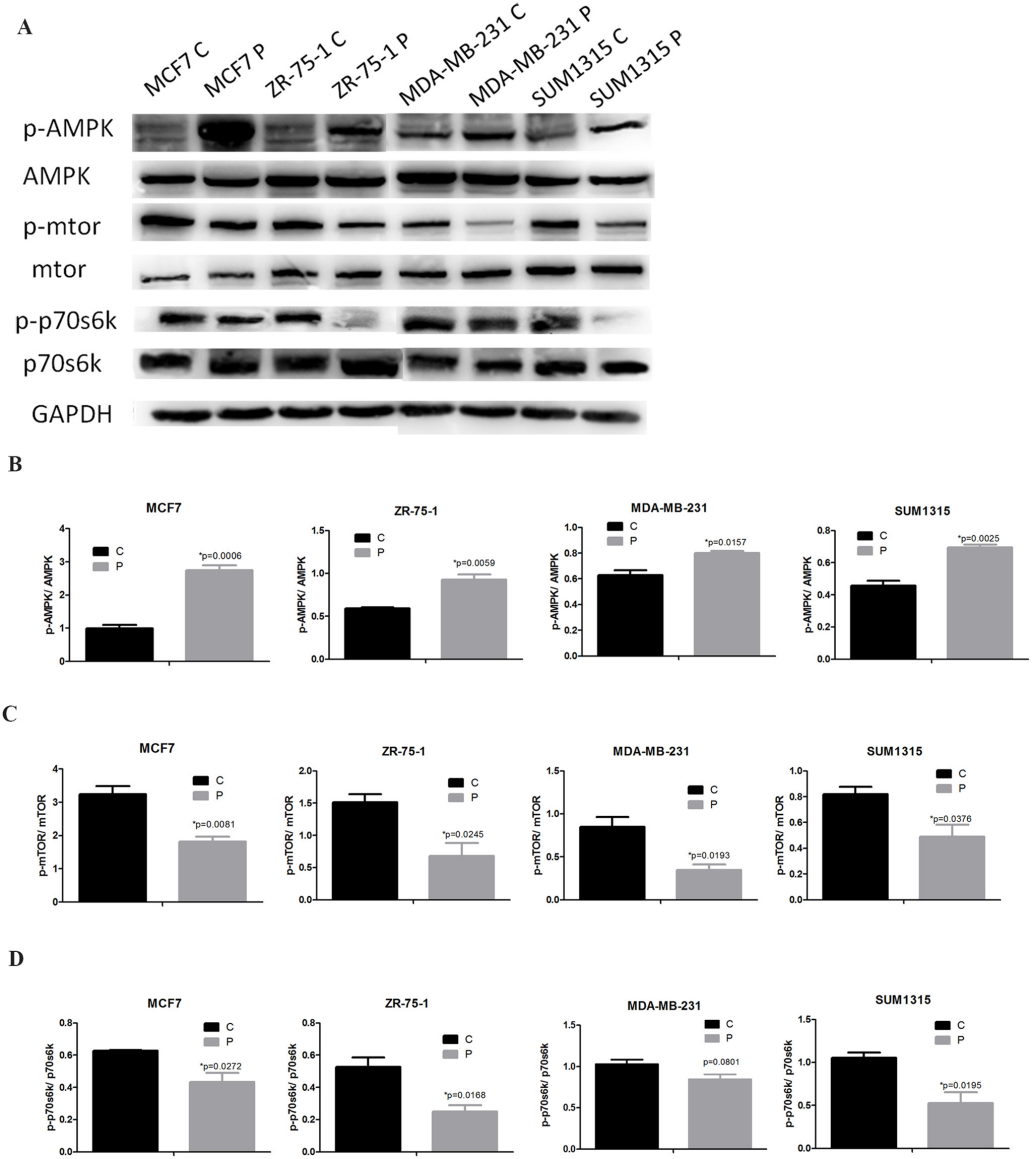
Activation of AMPK/mTOR/p70s6k signaling by phenformin. (A) MCF7, ZR-75-1, MDA-MB-231 and SUM1315 cells were treated with or without phenformin for 24 hours. Cell extracts were analyzed by western blotting to detect the expression of p-AMPK, AMPK, p-mTOR, mTOR, p-p70s6k, p70s6k and GAPDH. (B) Expression ratios analysis of p-AMPK to AMPK, (C) p-mTOR to mTOR, (D) p-p70s6k to p70s6k. The data are presented as mean±SEM of three replicates per group. Asterisks indicate significant differences at p<0.05 by Student’s t test. Phenformin-treated cells were labeled as P and control cells were labeled as C.

Western blotting was performed to determine whether AMPK, mTOR and p70s6k were involved in phenformin-induced apoptosis. Notably, Thr172 phosphorylation within the alpha subunit of AMPK significantly increased in the phenformin group compared with the control group after a 24-hour treatment. Moreover, downstream of AMPK, phosphorylation at Ser2448 of mTOR and at Thr389 of p70s6k decreased in response to phenformin compared with control. Meanwhile, there were no significant differences in total AMPK, mTOR or p70s6k expression between the control and phenformin-treated groups (Fig 5).

### Phenformin induces MET and inhibits the migration and metastasis of breast cancer cells

**Fig 6:**
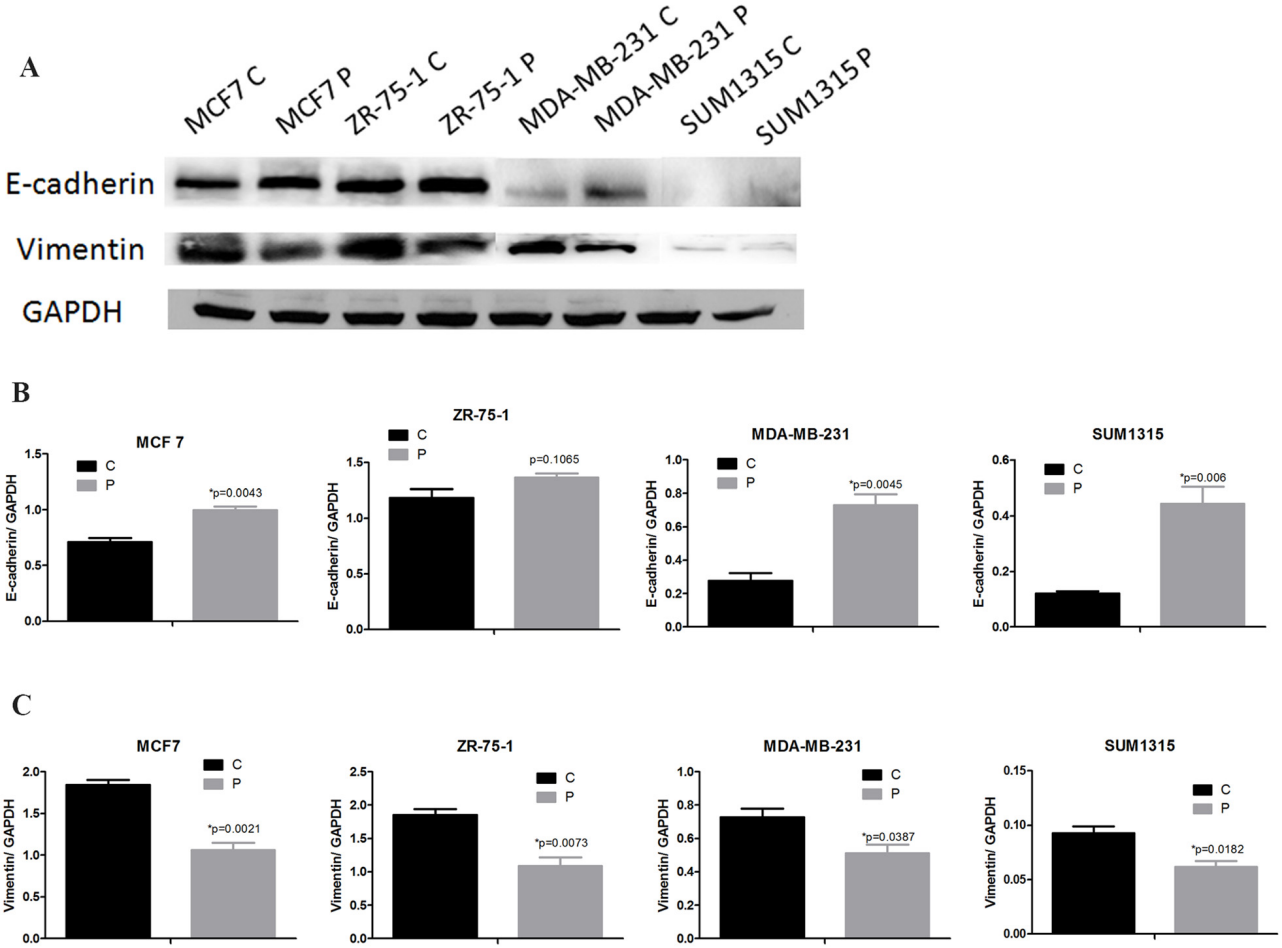
Phenformin induces epithelial features in breast cancer cells. (A) MCF7, ZR-75-1, MDA-MB-231 and SUM1315 cells were treated with or without phenformin for 24 hours. Cell extracts were analyzed by western blotting to detect the expression of E-cadherin, vimentin and GAPDH. (B) Expression ratios of E-cadherin to GAPDH, (C) vimentin to GAPDH. The data are presented as the mean±SEM of three replicates per group. Asterisks indicate significant differences at p<0.05 by Student’s t test. Phenformin-treated cells were labeled as P and control cells were labeled as C.

**Fig 7:**
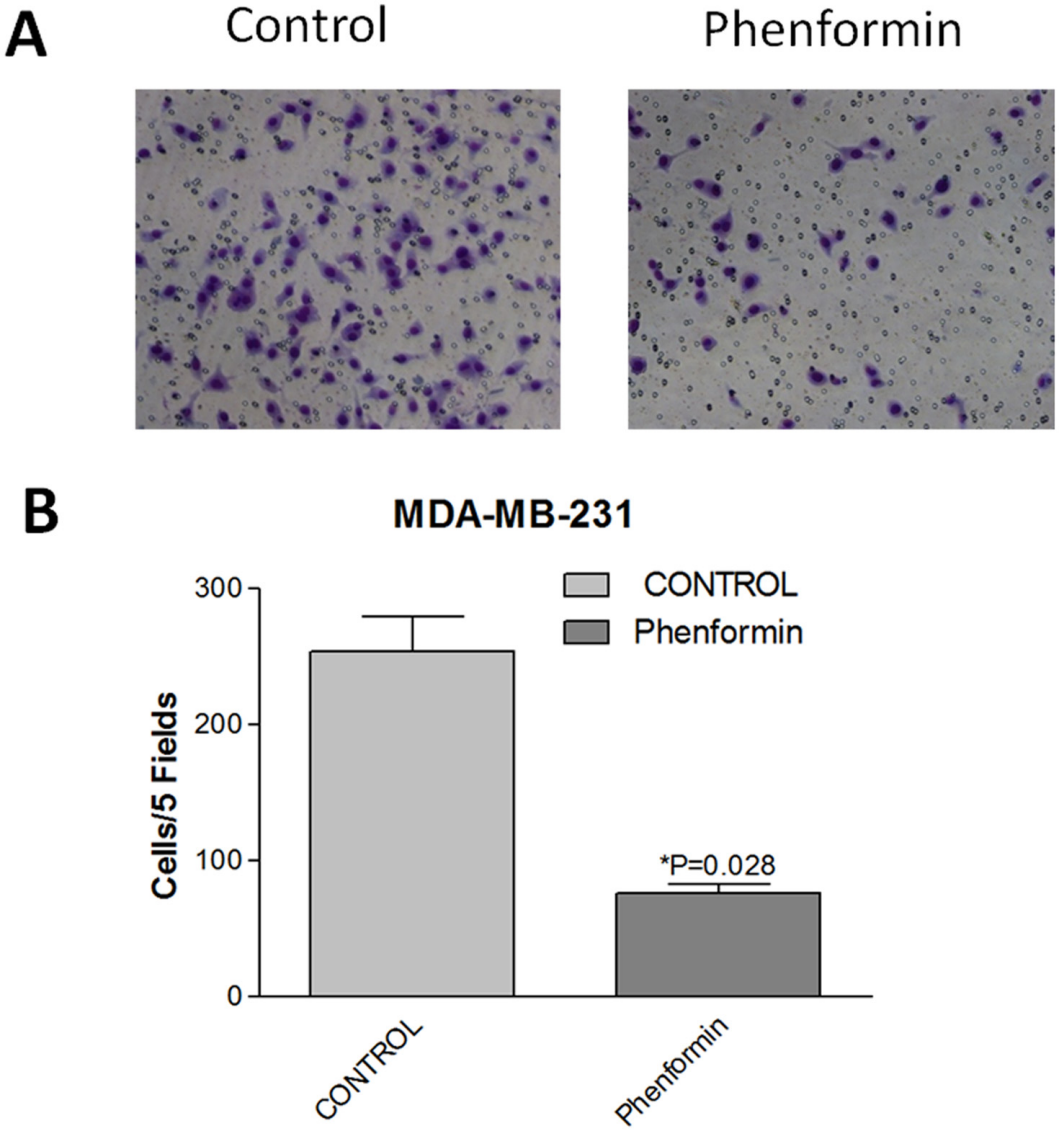
Phenformin inhibits MDA-MB-231 cells migration. (A) After incubation with phenformin for 24 hours, MDA-MB-231 cells (25,000 cells per chamber) were seeded in the upper chamber in serum free medium. The lower chamber contained medium with 10% FBS. After incubation for 16 hours, the cells were removed from the upper surface of the chamber membrane, and the cells on the lower surface of the chamber were stained with crystal violet and counted using a microscope(100X). (B) The number of cells/five fields was plotted. The data are presented as the mean±SEM of three replicates per group. Asterisks indicate significant differences at p<0.05 by Student’s t test.

**Fig 8:**
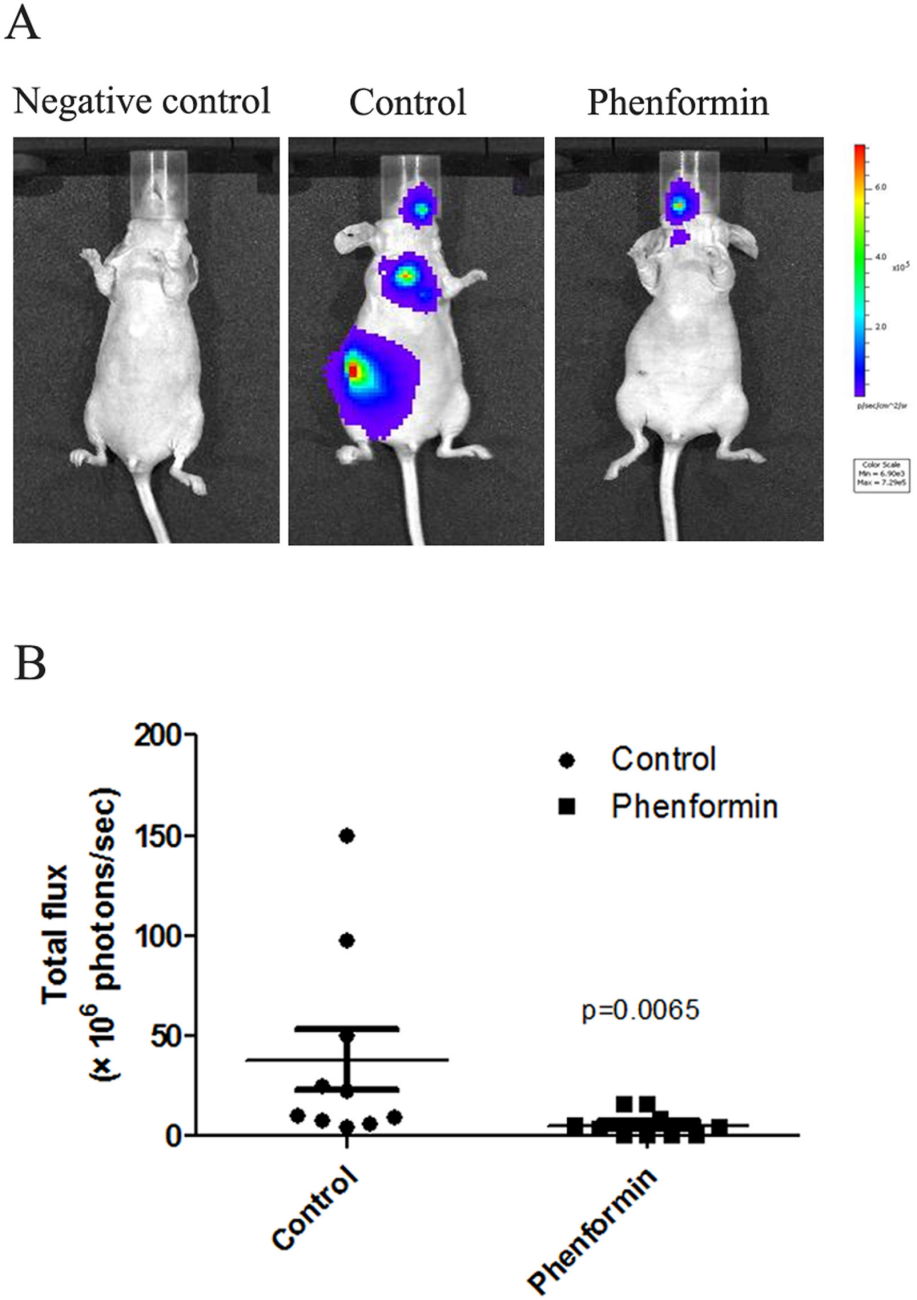
Phenformin inhibits the metastasis of MDA-MB-231 cells in nude mice. (A) The luciferase-tagged MDA-MB-231 cells were inoculated intracardially into female nude mice. After the injections, the mice were separated into the control and phenformin groups (10 mice per group). The control group’s drinking water was replaced with 5% sucrose. The phenformin group’s drinking water was replaced with 5% sucrose containing phenformin (300 mgkg^-1^). The development of metastasis was monitored using whole mouse fluorescence and bioluminescence imaging(negative control: mice did not receive an intracardiac injection of luciferase-expressing cells). (B)Four weeks after the intracardiac injections, the total flux signals in the control group were significantly higher than those in the phenformin group (p = 0.0065). The data are presented as the mean±SEM and the Wilcoxon rank sum test was used to identify significant differences in total flux between the control and phenformin- treated animals.

Phenformin treatment significantly increased the expression of the epithelial marker E-cadherin in MDA-MB-231 cells. Moderate changes in E-cadherin expression were observed in MCF-7, ZR-75-1 and SUM1315 cells. In contrast, the mesenchymal marker vimentin was significantly downregulated in all the cell lines after treatment with phenformin (Fig 6). Our findings indicated that phenformin might induce MET and prevent breast cancer metastasis. As the MDA-MB-231 cell line is the least sensitive of the four cell lines to phenformin-induced growth inhibition and the most aggressive among the four breast cancer cell lines, we utilized MDA-MB-231 cells to further evaluate the role of phenformin in breast cancer metastasis. Interestingly, the migration rate of phenformin-treated MDA-MB-231 cells was significantly decreased compared with control cells (Fig 7). Furthermore, four weeks after intracardiac injection of MDA-MB-231 cells, the total flux of the nude mice in the phenformin group was significantly lower than that in the control group (p = 0.0065) (Fig 8), which indicated that phenformin might inhibit breast cancer metastasis.

## Discussion

Recently, the anti-diabetic drug, metformin has attracted attention as an anti-cancer therapeutic for multiple types of cancers. Emerging evidence from retrospective population-based studies and preclinical studies have confirmed the anti-tumor activity of biguanides [15]. Metformin decreases tumor cell proliferation by improving insulin sensitivity and reducing hyperinsulinaemia. Moreover, metformin was reported to have a direct anti-tumor effect[16, 17]. Several studies in different models have demonstrated that phenformin, a member of the biguanides, is a more potent cancer inhibitor than metformin [9, 18]. Research on cancer prevention and treatment with biguanides has mainly focused on metformin; however, the mechanisms of action of phenformin related to cancer treatment remain elusive.

Phenformin was previously demonstrated to inhibit MCF7, T47D and MDA-MB-231 breast cancer cell proliferation [14]. In agreement with this finding, our results demonstrated the anti-cancer activity of phenformin in the MCF7, ZR-75-1, MDA-MB-231 and SUM1315 breast cancer cell lines: the respective IC_50_ values of phenformin in each cell line were 1.184±0.045 mM, 0.665±0.007 mM, 2.347±0.010 mM and 1.885±0.015 mM (mean± standard error). Consistent with a previous report on metformin, we found that MDA-MB-231 cells were the least sensitive of the four cell lines to the phenformin- mediated cell death [19].

Cell cycle dysregulation is a feature of tumor cells; therefore, cell-cycle arrest is a major indicator of anti-cancer activity [20]. Metformin was reported to inhibit MCF7 cell proliferation by promoting cell cycle arrest at G0/G1 [12], and AMPK-induced cell cycle arrest in hepatoma HepG2 cells has also been demonstrated [21]. Consistent with these observations, our results showed that phenformin exhibits strong anti-tumor activity by increasing the percentage of cells in G1 in all four breast cancer cell lines. As a cell cycle regulator, cyclin D1 is essential for the progression through G1 and is a candidate proto-oncogene [22]. In the current study, cyclin D1 expression significantly decreased in phenformin-treated breast cancer cells, which suggested that phenformin-induced cell cycle changes might be partly due to the downregulation of cyclin D1. There is controversy regarding the role of metformin in regulating cancer cell apoptosis. It was reported that metformin induced cell cycle arrest, but not apoptosis, in prostate cancer [23]. Nevertheless, others have shown that metformin increased the apoptosis of breast, colon and endometrial cancer cells [24–26]. Our results demonstrated that phenformin induces apoptosis in all four breast cancer cell lines. Our observations are consistent with a recent report that phenformin disrupted the mitochondrial membrane potential of breast cancer cells[14]. Death receptors and mitochondria represent the two main pathways that initiate apoptotic responses. Caspase 3 is the key executioner caspase in both pathways[27, 28]. In our study, cleaved caspase 3 levels increased in phenformin-treated ZR-75-1 and MDA-MB-231 breast cancer cells compared with control cells, which further confirmed that phenformin induced apoptosis in breast cancer cells.

AMPK is a well-known downstream molecule of metformin, and emerging evidence has indicated that metformin inhibits cancer cell proliferation by activating AMPK[12]. However, there is controversy regarding the role of AMPK in the anti-tumor activity of phenformin. It was reported that phenformin displays AMPK-independent effects on cancer cell proliferation and metabolism [29], whereas others have shown that the anti-tumor effect of phenformin is dependent, at least partially, on AMPK signaling [7, 10, 30]. Our results demonstrated that AMPK phosphorylation increases in breast cancer cells treated with phenformin. Furthermore, we are the first to report that phenformin not only activates AMPK but also inactivates mTOR and p70s6k, which act downstream of AMPK, suggesting that the AMPK/mTOR/p70s6k axis may be a major target of phenformin in inducing breast cancer apoptosis. The ERK signaling is important for the survival, proliferation and dissemination of human cancer cells [31]. Insulin receptor activation activates the ERK pathway; moreover, metformin was reported to inactivate ERK in breast cancer cells [12]. Consistent with this, we observed decreased p-ERK levels in phenformin-treated MCF7 and ZR-75-1 cells. However, p-ERK levels were unchanged in phenformin-treated MDA-MB-231 or SUM1315 cells, which might explain the less-sensitive nature of ERα-negative breast cancer to phenformin treatment.

The loss of cell-to-cell contacts and the gain of mesenchymal features, which define EMT, are crucial for cancer metastasis [32]. It was reported that metformin could reverse multidrug resistance and EMT [13]; moreover, mTORC2 activation induced EMT in mouse mammary epithelial cells [33]. Because mTOR inactivation was observed in breast cancer cells treated with phenformin, it was reasonable to investigate whether phenformin regulated EMT. Interestingly, our results indicated that phenformin induces MET in breast cancer, which suggested that phenformin might prevent breast cancer metastasis by reversing the EMT program. Consistent with this finding, migration assays confirmed the enhanced epithelial characteristics of phenformin-treated breast cancer cells. Phenformin was reported to inhibit both the development and growth of breast tumors in mice [7]; however, no data were available on the role of phenformin in breast cancer metastasis in mice. Our in vivo experiment demonstrated that phenformin significantly decreases the metastasis of MDA-MB-231 cells in mice, suggesting that phenformin might be a promising therapeutic for preventing breast cancer metastasis.

Phenformin has been withdrawn from use in the United States because of the increased incidence of lactate acidosis in elderly patients with renal failure compared with metformin[34], although it is still being used in certain other countries. Recently, the combination of 2-deoxyglucose with phenformin was reported to potentially eliminate the risk of lactic acidosis [11]. Moreover, phenformin possesses relatively lower toxicity compared with many commonly used chemotherapy regimens [10]. Taken together, phenformin might be more acceptable as a potent treatment for cancer than for diabetes.

Our study had limitations. To investigate the anti-tumor activity of phenformin in breast cancer cell lines of different genetic backgrounds, we utilized four breast cancer cell lines in most of the in vitro experiments. Because of the relatively large number of analyzed cell lines, most of the in vitro studies were performed at a single time point and a single concentration of phenformin. The short time frame of the experiments (24 hours) might not have been sufficient to reveal the effects of phenformin on cell proliferation, and the concentration range of phenformin (0-4mM) used in this study was relatively narrow. Thus, further studies are needed to confirm our results. Moreover, we only focused on the direct effects of phenformin on breast cancer cells. Further studies on the indirect effects of phenformin are warranted. Finally, further functional studies to prove a link between the changes of protein and migration ability induced by phenformin are needed to strengthen our results.

## Conclusions

In the current study, we demonstrated that phenformin induces cell death of MCF-7, ZR-75-1, MDA-MB-231 and SUM1315 cells, partly by modulating cell cycle and apoptosis. These effects were associated with the AMPK/mTOR/p70s6k and MAPK/ERK pathways. Furthermore, phenformin induced MET and decreased the metastasis of MDA-MB-231 cells in mice. Based on our findings and those of previous reports, phenformin may be an option for the treatment of breast cancer and the prevention of breast cancer metastasis.
